# Ethnobotanical study of medicinal plants used by people in Zegie Peninsula, Northwestern Ethiopia

**DOI:** 10.1186/1746-4269-3-12

**Published:** 2007-03-14

**Authors:** Tilahun Teklehaymanot, Mirutse Giday

**Affiliations:** 1 Endod and Other Medicinal Plants Unit, Aklilu Lemma Institute of Pathobiology, Addis Ababa University, P. O. Box 1176, Addis Ababa, Ethiopia

## Abstract

An ethnobotanical study was conducted from October 2005 to June 2006 to investigate the uses of medicinal plants by people in Zegie Peninsula, northwestern Ethiopia. Information was gathered from 200 people: 70 female and 130 males, using semistructured questionnaire. Of which, six were male local healers. The informants, except the healers, were selected randomly and no appointment was made prior to the visits. Informant consensus factor (ICF) for category of aliments and the fidelity level (FL) of the medicinal plants were determined. Sixty-seven medicinal plants used as a cure for 52 aliments were documented. They are distributed across 42 families and 64 genera. The most frequently utilized plant part was the underground part (root/rhizome/bulb) (42%). The largest number of remedies was used to treat gastrointestinal disorder and parasites infections (22.8%) followed by external injuries and parasites infections (22.1%). The administration routes are oral (51.4%), external (38.6%), nasal (7.9%), and ear (2.1%). The medicinal plants that were presumed to be effective in treating a certain category of disease, such as 'mich' and febrile diseases (0.80) had higher ICF values. This probably indicates a high incidence of these types of diseases in the region, possibly due to the poor socio-economic and sanitary conditions of this people. The medicinal plants that are widely used by the local people or used as a remedy for a specific aliment have higher FL values (*Carissa spinarum*, *Clausena anisata*, *Acokanthera schimperi*, *Calpurnia aurea, Ficus thonningii*, and *Cyphostemma junceum*) than those that are less popular or used to treat more than one type of aliments (*Plumbago zeylanicum, Dorstenia barnimiana*).

## Background

Ethnobotanical studies are often significant in revealing locally important plant species especially for the discovery of crude drugs. Right from its beginning, the documentation of traditional knowledge, especially on the medicinal uses of plants, has provided many important drugs of modern day [1,2]. Traditional medicine still remains the main resource for a large majority (80%) of the people in Ethiopia for treating health problems and a traditional medical consultancy including the consumption of the medicinal plants has a much lower cost than modern medical attention [3-5].

Out of the total flowering plants reported from the world, more than 50,000 are used for medicinal purposes [6,7]. In Ethiopia, about 800 species of plants are used in the traditional health care system to treat nearly 300 mental and physical disorders. The wide spread use of traditional medicine among both urban and rural population in Ethiopia could be attributed to cultural acceptability, efficacy against certain type of diseases, physical accessibility and economic affordability as compared to modern medicine. Ethiopian traditional medical system is characterized by variation and is shaped by the ecological diversities of the country, socio-cultural background of the different ethnic groups as well as historical developments, which are related to migration, introduction of foreign culture and religion. Previous studies showed the existence of traditional medical pluralism in the country. In Ethiopia, either the knowledge from herbalists is passed secretively from one generation to the next through words of mouths or their descendants inherit the medico-spiritual manuscripts [8-12].

The study of Ethiopian medicinal plants has not been realized as fully as that of India or other traditional communities elsewhere [13]. In Ethiopia, though there has been some organized ethnomedicinal studies, there is limited development of therapeutic products and the indigenous knowledge on usage of medicinal plants as folk remedies are getting lost owing to migration from rural to urban areas, industrialization, rapid loss of natural habitats and changes in life style. In addition, there is a lack of ethnobotanical survey carried out in most parts of the country. In view of these, documentation of the traditional uses of medicinal plants is an urgent matter and important to preserve the knowledge. Furthermore, most of the ethnomedicinal studies in northern part of Ethiopia are focused on 'Medihanit Awakie' (professional traditional practitioners) and the ancient medico-magical and/or medico-spiritual manuscripts and old Gee'z manuscripts [11,14,15], and ignore the knowledge of ordinary people in the locality [16]. Thus, the purpose of this study is to investigate the traditional uses of medicinal plants by the ordinary people in Zegie Peninsula and to provide baseline data for future pharmacological and phytochemical studies.

## Methods

### Description of the Study Area

**Figure 1:**
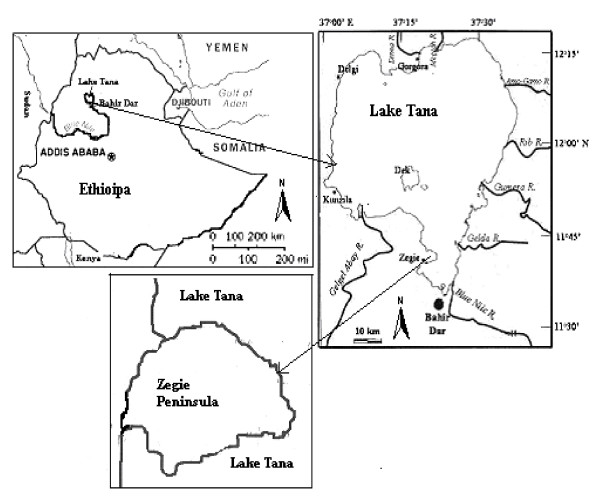
Map of Zegie Peninsula in Ethiopia.

Zegie Peninsula (11° 43' N, 37° 20' E) is located at 600 km northwest of Addis Ababa in the country's northwest highlands, at an altitude of approximately 1800 meters. It is partly surrounded by Lake Tana, which is the largest lake in Ethiopia and the source of the Blue Nile. Zegie Peninsula is about three hours motorboat drive or 37 km on land from Bahir Dar, the capital city of Amahra Regional State (Fig. 1). The residents are Amahra people and speak the country's official language Amharic. Tankwas (papyrus boats) of ancient design, manufactured on the shores of Lake Tana, are the alternative forms of transport for the local people between Zegie and Bahir Dar. There are seven monasteries on the peninsula from the 16th and 17th century. Ura Kidane Mhret, one of the monasteries, houses myriads of treasures, beautiful mural paintings, icons, scrolls and thousand-year-old manuscriptsas well as crowns and dresses from Ethiopian Emperors. During the study time, there were no modern health facilities in the area. The main occupation of the people is fishing, and coffee plantation. Until recently, there was no farming practice because the monasteries in the peninsula had forbidden the use of any type of draft animal for farming. Nevertheless, currently, the people have started farming and clearing the forest for agricultural purposes and this may affect the natural habitats of some of the medicinal plants.

### Survey on the Use of Medicinal Plants

The ethnobotanical surveys were carried out from October 2005 to June 2006 using semistructured questionnaire [17] and interview was conducted in Amharic. Prior to the administration of the questionnaire, conversations with the informants were held with the assistance of local Farmers' Association representative to elaborate the objective of the study and to build on trust with the common goal to document and preserve the knowledge on medicinal plants. Two hundred informants were interviewed out of about 2855 inhabitants (1,338 females and 1517 males) of the Zegie peninsula (unpublished data, Bahir Dar Zuria Woreda Administration), these included 130 males and 70 females. Of which, six were male local healers (the only ones found on the peninsula). The female informants' age ranges from 30 to 85 years and the mean age is 51 years, and the male informants' age ranges from 30 to 93 years and the mean age is 64 years. The informants, except the healers, were selected randomly and no appointment was made prior to the visits. They were asked to give their knowledge about the plants they use against a disease, plant parts harvested, method of preparation of the remedy, details of administration and the dosage. Specimens of the reported medicinal plants were collected during regular systematic walk in the fields and identified by specialists at the Aklilu Lemma Institute of Pathobiology and the National Herbarium of Addis Ababa University following the Flora of Ethiopia and Eritrea [18-21]. Voucher specimens were deposited at the Herbarium of Aklilu Lemma Institute of Pathobiology, Addis Ababa University.

### Data Analysis

The reported aliments were grouped into 10 categories based on the information gathered from the interviewees. The categories were: evil eye and 'satan beshita' (devil sickness), external injuries and parasites infections, gastrointestinal disorder and parasites infections, 'mich' (febrile disease characterized by fever, headache, sweating, *Herpes labialis*, and muscle spasm) and febrile diseases, rabies and internal disease, respiratory and throat infections, sensorial disease, snake bite, swelling (non-infectious or infectious swelling) and cancer, and venereal disease and impotence. Informant consensus factor (ICF) was calculated for each category of aliments to identify the agreements of the informants on the reported cures for the group of aliments. ICF was calculated as follows: number of use citations in each category (n_ur_) minus the number of species used (n_t_), divided by the number of use citations in each category minus one [22].

ICF=nur−ntnur−1
 MathType@MTEF@5@5@+=feaafiart1ev1aaatCvAUfKttLearuWrP9MDH5MBPbIqV92AaeXatLxBI9gBaebbnrfifHhDYfgasaacH8akY=wiFfYdH8Gipec8Eeeu0xXdbba9frFj0=OqFfea0dXdd9vqai=hGuQ8kuc9pgc9s8qqaq=dirpe0xb9q8qiLsFr0=vr0=vr0dc8meaabaqaciaacaGaaeqabaqabeGadaaakeaacqqGjbqscqqGdbWqcqqGgbGrcqGH9aqpdaWcaaqaaiabb6gaUnaaBaaaleaacqqG1bqDcqqGYbGCaeqaaOGaeyOeI0IaeeOBa42aaSbaaSqaaiabbsha0bqabaaakeaacqqGUbGBdaWgaaWcbaGaeeyDauNaeeOCaihabeaakiabgkHiTiabigdaXaaaaaa@3FB7@

The fidelity level (FL), the percentage of informants claiming the use of a certain plant for the same major purpose, was calculated for the most frequently reported diseases or ailments as:

FL(%)=NpN×100
 MathType@MTEF@5@5@+=feaafiart1ev1aaatCvAUfKttLearuWrP9MDH5MBPbIqV92AaeXatLxBI9gBaebbnrfifHhDYfgasaacH8akY=wiFfYdH8Gipec8Eeeu0xXdbba9frFj0=OqFfea0dXdd9vqai=hGuQ8kuc9pgc9s8qqaq=dirpe0xb9q8qiLsFr0=vr0=vr0dc8meaabaqaciaacaGaaeqabaqabeGadaaakeaacqqGgbGrcqqGmbatcqGGOaakcqGGLaqjcqGGPaqkcqGH9aqpdaWcaaqaaiabb6eaojabbchaWbqaaiabb6eaobaacqGHxdaTcqaIXaqmcqaIWaamcqaIWaamaaa@3B08@

Where Np is the number of informants that claim a use of a plant species to treat a particular disease, and N is the number of informants that use the plants as a medicine to treat any given disease [23]. These two methods are helpful in the selection of plants for further studies.

## Result and discussion

### Knowledge of Informants and Medicinal Plants

Eighty two percent of informants reported remedies for 52 aliments. Of which 26% are females and 74% are males, which indicated that most people continue to use traditional systems of health care including medicinal plants alone or in combination with modern pharmaceuticals. This continued reliance of many African people on traditional medicines is partly due to economic circumstances, which place modern health facilities, services and pharmaceuticals out of the reach of the majority of the population. However, in many cases, it is also attributable to the widespread belief in the effectiveness of many traditional therapies. Even where western biomedical care is available, many people still prefer traditional treatments for treating many aliments [4,5,11,24].

The females reported remedies to diseases associated to children such as 'mich', stomachache, 'kuruba' (diarrhea, dysentery, stomach disorder), dysentery, tonsillitis and babies' sickness (thinning, loss of appetite). The males reported (mean = 6.7 ± 2.79) more number of remedies than the females (mean = 2.3 ± 0.9) and there is a significant difference (p = 0.004) between female and male and agrees with the previous reports of ethnobotanical studies in northern and southern Ethiopia [4,5]. This is because the traditional knowledge in the family or community is passed from male parent to his first-born son [25,26].

All the healers were male and the number of aliments reported by them ranged from six to twenty. They also reported combination of multiple medicinal plants to treat an illness, whereas most of the non-healers, both females and males reported only a single medicinal plant treatment (Table 1, 2). The multiple prescriptions reported by the healers usually contain a range of pharmacologically active compounds; in some cases, it is not known which ingredients are important for the therapeutic effect and some are used as adjuvants [27].

**Table 1:** Single medicinal plants treatment with parts used and preparation

**Species**	**Family**	**Local Name**	**Use(s)**	**Parts used and preparation**
*Achyranthes aspera *L.	Amaranthaceae	Telenzje	'shererit kusil' (*Herpes zoster*)	Chewing fresh leaves
			blood clotting	Dressing with crushed fresh leaves
*Acokanthera schimperi *(A. DC.) Schweinf.	Apocynaceae	Yemerz Enchet	'kusil'	Dressing with crushed whole plant
			'yetat merz' (bacterial infection of nail)	Dressing with crushed fresh root
*Allium sativum L.*	Alliaceae	Nech Shinkurt	'ayne maz' (eye sickness)	Rubbing with warmed bulb
			evil eye	Smelling aroma of bulb
*Asparagus africanus *Lam.	Asparagaceae	Yeset Kest	'sinfete wesib'	Root powder is eaten with chicken soup
*Brucea antidysenterica *J. F. Mill.	Simaroubaceae	Aballo (Waginos)	'bullad' (weight loss fever, itching, diarrhea)	Fruit powder mixed with honey and fermented for seven days is taken orally until cure
			'fintita sigelebet' (Haemorrhoids)	Fruit powder mixed with milk is taken orally for three days
			'mushuro' (weight loss, dysentery and fever)	Root powder mixed with honey is taken orally until cure
			dysentery	Juice of leaf is taken orally in the morning
			'chiffea' (Eczema)	Dressing with inner bark paste mixed with butter or oil
*Calpurnia aurea *(Alt.) Benth.	Fabaceae	Digita	'kuruba'	Leaves or Fruit powder mixed with water or honey is taken orally
*Carica papaya *L.	Caricaceae	Papaya	malaria	Juice of leaves is taken orally
*Centella asiatica *L.	Apiaceae	Yeayit Joro	swelling	Dressing with leaf paste
*Clausena anisata *(Willd.) Benth	Rutaceae	Limche	ear sickness	Juice of leaves is used as ear drop
*Clausena anisata *(Willd.) Benth	Rutaceae	Limche	stomachache	Chewing root
*Clematis hirsuta *Perr & Guill	Ranunculaceae	Azo Hareg	'mich'	Juice of fresh leaves is used as body lotion
			cough	Juice of leaves with butter of fat is taken orally
			swelling	Dressing with Leaf paste
*Commelina sp.*	Commelinaceae	Yemariam Wuha	allergic	Dressing with crushed fresh leaf
			ear infection	Juice of leaves as ear drop
*Croton marcostachyus *Del.	Euphorbiaceae	Bissana	'ekeke' (scabies)	Dressing with Crushed leaves mixed with butter or oil
			'kuruba'	Leaves are eaten with wat(Diarrhoea, dysentery, stomach disorder) (local soup)
			'wef beshita' (hepatitis, jaundice)	Leaf powder mixed with water is taken orally for seven days
			diarrhea	Leaf powder mixed with water is taken orally
			quaqucha (*Tinea versicolor*)	Rubbing and dressing with Latex from leaves
*Cucumis ficifolius *A. Rich.	Curcurbitaceae	Yemidir Embuay (Este Melecot)	'ayn bar tessa'	Chewing root
			'majrat getr' (meningitis)	Root powder mixed with honey taken orally
			'nessr' (epistaxis)	Juice of root applied though nose
			'wef beshita'	Root powder is taken mixed with skimmed milk or noug orally in the morning
			rabies	Root powder is eaten with tef kita
			stomachache, 'kuruba', umbilical cord labouring	Chewing root
*Cussonia holstii *Harms ex. Engl.	Araliaceae	Sila	burning	Dressing with crushed fresh leaves
*Cyphostemma junceum *(Webb) Decoings ex Wild & Drummond	Vitaceae	Etse Zewe	snake bite	Chewing roots
*Datura stramonium*	Solanaceae	Astenagir	swelling	Dressing with leaf paste
			tooth ache	Fresh leaves are boiled with water and the vapour is inhaled
			'fore fore' (dandruff)	Fresh leaves are used for rubbing and dressing
			'kusil'	Dressing with leaf paste
*Dorstenia barnimiana *Schwienf.	Moraceae	Work Bemeda	'wef beshita'	Root powder is taken with skimmed milk or noug orally in the morning
			'yeahya kintarot' (donkey's wart)	Dressing with root paste
			cancer	Making small opening and inserting the root
			rabies	Root powder is taken with skimmed milk or noug orally in the morning for seven days
			syphilis	Root powder is taken with honey orally in the morning
			weight loss, diarrhea and fever	Root powder mixed honey and fermented for seven days is taken orally in the morning until cured
*Draceana steudeneri *Engl.	Dracaenaceae	Etse Patos	evil eye	Root is burned and smoke is inhaled
*Echinops kebericho *Mesfin	Compositae	Kebercho	evil eye	Root powder is sprinkled on burning charcoal and smoke is inhaled
*Euphorbia abyssinica *J. F. Gmel.	Euphorbiaceae	Qulqwal	venereal diseases	Latex is eaten with tef of wheat kit
			'wef beshita'	Latex mixed with water is taken orally
			rabies	Root powder mixed water is taken orally
*Euphorbia tirucalli *L.	Euphorbiaceae	Kinchib	'kintarot'	Rubbing with latex and dressing
			'kusil'	Dressing with latex
*Ferrula communis *L.	Apiaceae	Dog	cough	Filtrate of boiled root mixed with honey taken orally until cured
*Ficus thonningii *Blume.	Moraceae	Chibha	'ayn bar tessa' (lose of appetite)	Root with Noug is eaten
			diarrhea	Chewing root
			stomachache	Chewing inner Bark
*Glinus lotoides *L.	Molluginaceae	Meterea	tapeworm	Fruit powder mixed with noug is taken orally
*Gnidia glauca *(Fresen)	Thymelaeaceae	Beto	rabies	Root powder mixed with skimmed milk is taken orally for seven days
*Gossypium herbaceum *L.	Malvaceae	Tit	snake bite	Chewing root
*Hagenia abyssinica *(Bruce) J. F. Gmel.	Rosaceae	Kosso	'kosso' (tape worm)	Powder mixed with water and fermented over night is taken orally in the morning
*Helinu mystacinus *(Ait.) E. Mey. ex Steud	Rhamnaceae	Esat Abered	burning	Dressing with crushed fresh leaves
*Huernia concinna *N. E. Br.	Asclepiadaceae	Yelam Tute	'kusil', swelling	Dressing with crushed fresh leaf
*Impomea *sp.	Convolvulaceae	Filatsut	babies' sickness	Bathing with crushed leaf and stem
			cancer	Making small opening and inserting the root
*Indigofera spicata *Forssk.	Fabaceae	Yebab Alenga	babies' sickness	Bathing with crushed fresh leaf and stem
			stomachache	Chewing root
*Justicia schimperiana *(Hochst. ex A. Nees) T. Anders	Acanthaceae	Sensel (Smiza)	'wef beshita', 'kuruba'	Juice of leaves is taken orally
			evil eye	Smelling the aroma of fresh root
*Kalanchoe petitana *A. Rich.	Crassulaceae	Endehuahula	swelling	Making small opening and inserting the root
*Millettia ferruginea *(Hochst.) Bark	Fabaceae	Birbira	'mujelea' (chigger)	Dressing with fruit paste mixed with butter
			'tfre metmte' (bacterial infection of nails)	Dressing with leaf paste
			'yejoro kunkun' (earache)	Juice of leaves or stem is used as ear drop
			amoeba	Fruits powder mixed with honey is taken orally
*Mimusops kummel *Bruce ex. DC.	Sapotaceae	Eshe	amoeba	Eating fruits
*Momordica foetida *Schumach	Cucurbitaceae	Qura Hareg	'zuresh' (babies sickness)	Bathing with crushed fresh root
*Myrtus communis *L.	Myrtaceae	Ades	'fore fore' (Dandruff)	Bathing with crushed fresh leaves
			diarrhea, stomach disorder	Juice of leaf is taken orally in the morning
*Ocimum lamiifolium *Hochst.	Lamiaceae	Dama Kesse	'kusil'	Fresh crushed leaves dressing
*Ocimum lamiifolium *Hochst.	Lamiaceae	Dama Kesse	'kusil'	Dressing with Bark paste
			'mich'	Juice of leaves is taken with coffee orally
*Pergularia daemia *L.	Asclepiadaceae	Yeayit Hareg	snake bite	Making small cut at location and inserting root
*Phytolacca dodecandra *L'Herit	Phytolaceae	Endod (Male)	'kuruba'	Root or leaf powder mixed with water is taken orally
			'kusil'	Dressing with Fruit paste
			'wef beshita'	Leaf powder mixed with water is taken orally
			rabies	Root paste is taken with tef kita in the morning for seven days
*Plumbago zeylanicum *L.	Plumbaginaceae	Amira	'kurtimat' (rheumatic Pain)	Fresh leaves are boiled and the filtrate is taken with honey orally for seven days
			cancer	Root powder mixed with digne (sulphur) is applied
			cough	Fresh leaves are boiled and the filtrate is taken with fermented butter orally
			snake bite	Chewing Leaves
			swelling	Dressing with root paste
*Podocarpus gracilis*	Podocarpaceae	Zigba	vomiting	Juice of leaves is taken orally
*Rhamnus prinoides *L.	Rhamnaceae	Gesho	'chiffea' (Eczema)	Appling leaf paste mixed with butter as ointment
*Ricinus communis *L.	Euphorbiaceae	Kachima	'kuruba'	Juice of root is taken orally
			tooth ache	Chewing fresh root
*Rumex nepalensis *Spreng.	Polygonaceae	Tult	'entil siwerd' (tonsillitis), 'kuruba'	Juice of root is taken orally
			umbilical cord labouring	Tying fresh root around west
*Ruta chalepensis *L.	Rutaceae	Tena Adam	evil eye	Smelling aroma of fresh leaf and stem
			flue	Juice of leaves is taken with coffee
*Sansevieria erythraeae *Mattei	Dracaenaceae	Algeti/cheret	'sinfete wesib' (impotence)	Root powder is taken with tef potage
*Sida ternata *L. F.	Malvaceae	Yemidir Hareg	'lashet' (fungal disease)	Dressing with crushed fresh leaves
*Solanum marginatum *L.f	Solanaceae	Geber Embuay	'kusil', swelling	Dressing with crushed fresh root
*Stephania abyssinica *(Dillon. & A. Rich.) Walp.	Menispermaceae	Kib Kitel (Etse Eyesus)	'kuruba'	Juice of root is taken orally
			babies' sickness	Juice of leaves mixed with butter is taken orally
			stomachache	Juice of leaf and stem is taken orally
			'kintarot'	Dressing with stem paste
			'girfita' (fever, headache)	Bathing with crushed fresh leaves
*Stereospermum kunthianum*	Bignoniaceae	Zana	'kola kusil' (infected cut or wound)	Dressing with Bark paste
*Taverniera abyssinica *A. Rich	Fabaceae	Dingetegna	Vomiting, dysentery	Chewing root
*Verbascum sinaiticum *Benth.	Scrophulariaceae	Daba Keded	'kusil'	Dressing with Fresh crushed leaves
			diarrhea, stomachache	Juice of root is taken orally
*Verbena officinalis *L.	Verbenaceae	Atuch	'gusmit' (stomach disorder)	Juice of leaves is taken orally
			'yeshererit beshita' (*Herpes zoster*)	Dressing with leaf paste
			ear sickness	Juice of fruit with olive oil is used as ear drop
			evil eye	Smelling of aroma of fresh root
			snake bite	Chewing root
			stomachache	Chewing root
			'wesfat' (ascaris)	Juice of root is taken orally
*Vernonia adoensis *Sch. Bip. ex Walp.	Asteraceae	Este Mossa	menstrual disorders	Root are chewed with honey
*Vernonia amygdalina *Del.	Asteraceae	Girawa	'entil siwerd' (Tonsillitis)	Juice of leaf is taken orally
			'likift' (devil sickness, madness)	Root is burned and smoke is inhaled
			'satan beshita' (devil sickness)	Bathing with crushed fresh leaves
			evil eye, 'satan beshita', 'tesbo beshita' (epidemic disease)	Root powder is sprinkled on burning charcoal and smoke is inhaled
*Ximenia americana *L.	Olacaceae	Enkoye	'entil siwerd' (tonsillitis)	Juice of bark is taken orally
			'kusil'	Dressing with bark paste
*Zehneria scabra*	Asteraceae	Hareg Ressa (Este Sabek, Shahirit)	'mich'	Leaves and stem are boiled and the vapour is inhaled and bathing
			'kintarot' (wart)	Pressing with warmed stem
*Zingiber officinale *Rosc.	Zingiberaceae	Zinjible	stomachache	Chewing rhizome

**Table 2:** Multiple medicinal plants treatment with parts used and preparation

**Species**		**Family**	**Local name**	**Use(s)**	**Parts used and preparation**
1	*Pavonia urens *Cav.	Malvaceae	Ablalit	'sinfete wesib' (impotence)	Root powder taken with tella (local beverage) orally
2	*Asparagus africanus *Lam.	Asparagaceae	Set Kest		
3	*Ferrula communis *L.	Apiacae	Dog (Ramiron)		
4	*Clerodendrum myricoides *(Hochst.) Vatke	Verbenaceae	Misrich		
1	*Carissa spinarum *L.	Apocynaceae	Agam	evil eye	Sprinkling root powder on burning charcoal and inhaling smoke
2	*Capparis tomentosa *Lam.	Capparidaceae	Gumero		
3	*Verbascum sinaiticum *Benth.	Scrophulariaceae	Daba Keded		
4	*Achyranthes aspera *L.	Amaranthaceae	Telenzje		
5	*Justicia schimperiana *(Hochst. ex A. Nees) T. Anders	Acanthaceae	Sensel (Smiza)		
1	*Carissa spinarum *L.	Apocynaceae	Agam	evil eye	Sprinkling root powder on burning charcoal and smoke inhaled
2	*Capparis tomentosa *Lam.	Capparidaceae	Gumero		
3	*Asparagus africanus *Lam.	Asparagaceae	Set Kest		
4	*Clausena anisata *(Willd.) Benth	Rutaceae	Limchi		
5	*Draceana steudeneri *Engl.	Dracaenaceae	Etse Patos		
6	*Justicia schimperiana *(Hochst. ex A. Nees) T. Anders	Acanthaceae	Senel		
7	*Echinops kebericho *Mesfin	Asteraceae	Kebercho		
8	*Ruta chalepensis *L.	Rutaceae	Tena Adam		
9	*Allium sativum *L.	Alliaceae	Nech Shnkurt		
1	*Carissa spinarum *L.	Apocynaceae	Agam	evil eye	Root paste with water taken orally
2	*Capparis tomentosa *Lam.	Capparidaceae	Gumero		
3	*Clausena anisata *(Willd.) Benth	Rutaceae	Limchi		
1	*Croton marcostachyus *Del.	Euphorbiaceae	Bissana	stomachache disorder	Leaves, root and seeds boiled in butter taken orally
2	*Solanum indicum *L.	Solanaceae	Nech Embuay		
3	*Eragrostis tef *(Zucc.) Trotter	Poaceae	Tef		
1	*Brucea antidysenterica *J. F. Mill.	Simaroubaceae	Aballo (Waginos)	'chiffea'	Dressing root paste with honey
2	*Cucumis ficifolius *A. Rich.	Cucurbitaceous	Yemidir Embuay		
1	*Brucea antidysenterica *J. F. Mill.	Simaroubaceae	Aballo	craziness	Bathing with crushed fresh leaves and root
2	*Podocarpus gracilis*	Podocarpaceae	Zigba		

The number of ethnomedicinally important plant species documented in Zegie Peninsula was 67. These species belong to 64 genera and 44 families. The genera Asteraceae, Euphorbiaceae, Fabaceae and Solanaceae were families with four species each followed by Malvaceae with three species and, Apocynaceae, Asclepiadaceae, Cucurbitaceae, Dracaenaceae, Moraceae, Rhamnaceae and Rutaceae, each contributing two species.

**Figure 2:**
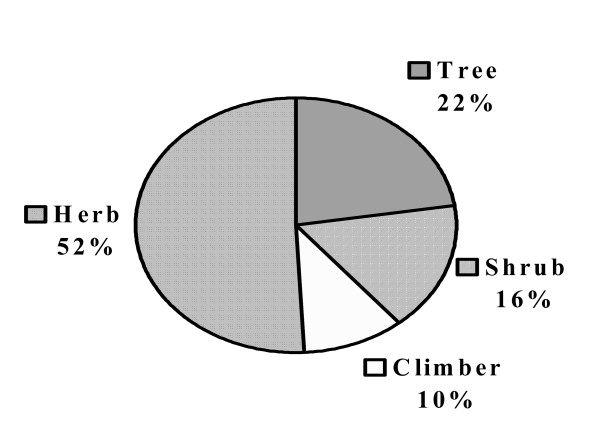
Percentages of habits of medicinal plants.

The most frequently utilized plant part was the underground part (root/rhizome/bulb = 42%) (Table 4). In studies conducted in Ethiopia, root (58.3%) is one of the most extensively used plant part in preparation of traditional herbal medicine [11]. In this study, herbs are used predominantly (52%, Fig. 2) as in most part of Ethiopia (34.8%) [27,28].

**Table 4:** Frequency of plant parts used for the preparation of remedies

Plant parts used	Number of medicinal plant species	Percentage
Leaf	53	37%
Root	58	40%
Flower	10	7%
Leaf/Stem	4	3%
Leaf/Root	3	2%
Bark	6	4%
Latex	4	3%
Rhizome	1	1%
Bulb	2	1%
Seed	1	1%
Stem	2	1%
Whole	1	1%

The largest number of remedies was used to treat gastrointestinal disorder and parasites (22.8%) followed by external injuries and parasites (22.1%), rabies and internal diseases (17.9%). The proportion of remedies used for treatment of gastrointestinal related disease are also high in most studies conducted in Ethiopia, accounting for 35% compared to other type of remedies that were compiled as being used against human aliments [28]. The rest were used to treat swelling and cancer (8.3%), evil eye and devil sickness (6.2%), sensorial disease (6.2%), venereal disease and impotence (4.8%), 'mich' and febrile diseases (4.1%), respiratory and throat infection (4.1%), and snake bite (3.4%). Multiple plants treatments with different combinations of medicinal plants were used to treat seven external and internal illnesses. Seventy eight percent of the multiple plants treatments were roots and were prepared by mixing the ingredients with different proportions. Three were used to treat evil eye and one of the poly-herbal remedy had nine medicinal plants (Table 3).

**Table 3:** Medicinal plants of veterinary importance with parts used and preparation

**Species**	**Family**	**Local name**	**Habit**	**Use(s)**	**Preparation**
*Achyranthes aspera *L.	Amaranthaceae	Telenzje	Herb	blood clotting	Dressing with crushed leaves
*Calpurnia aurea *(Alt.) Benth.	Fabaceae	Digita	Tree	dysentery	Leaf paste mixed with water is applied orally
*Croton marcostachyus *Del.	Euphorbiaceae	Bissana	Tree	'wef beshita'	Making small opening and inserting crushed leaves with salt and soot in the opening
*Cyphostemma junceum *(Webb) Decoings ex Wild & Drummond	Vitaceae	Etse Zewe	Climber	snake bite	Crushed fresh root is applied orally
*Ficus thonningii *Blume.	Moraceae	Chibha	Tree	stomach disorder	Crushed fresh root is applied orally
*Ocimum lamiifolium *Hochst.	Lamiaceae	Dama Kesse	Shrub	'mich'	Juice of leaves with Dagusa injera is applied orally
*Phytolacca dodecandra *L'Herit	Phytolaceae	Endod (Male)	Shrub	'wef beshita'	Crushed fresh leaves is applied orally
*Plumbago zeylanicum *L.	Plumbaginaceae	Amira	Herb	swelling	Dressing with root paste

### Route and dosage of administration

The administration routes are oral (51.4%), external (38.6%), nasal (7.9%), and through the ear (2.1%). The remedies are taken with water, skimmed milk, honey, tef injera (local thin bread made from tef, *Eragrostis tef*) and boiled coffee. The measurements used to determine the dosages are not standardized and depend on the age and physical appearance of the patient, sociocultural explanation of the illness, diagnosis and experience of individual herbalist [5,11]. Children are given less than adults, such as, one fourth of a coffee cup (2 ml to 5 ml), whereas, an adult is given up to one glass (approximately 250 ml) depending on the type of illness and treatment. The quantity of plant part used is measured by number of leaves, seeds and fruits, and length of root. For example, seven young leaves of *Justicia schimperiana *are used to treat ascaris, seven seeds of *Calpurnia aurea *are used to treat diarrhea and about 2 cm of root of *Dorstenia barnimiana *is used to treat cancer. The frequency of treatment depends on the type of illness and severity. In preparation of poly-herbal medicines, each medicinal plant is dried, powdered and stored separately, and the amount taken from each for any given disease varies.

### Veterinary Important Traditional Medicines

Eight species of medicinal plants have veterinary importance. The plant parts used were leaf (62.5%) and root (37.5%). These are used as remedy for seven internal and external illnesses (Table 3). The number of veterinary important medicinal plants is low compared to those areas with culture of cattle raring. Giday and Ameni [29] documented 83 medicinal plants that are used to treat 37 types of livestock aliments. In our study area, people are not accustomed to cattle raring and, therefore, have low knowledge of veterinary important medicinal plants.

### Informants consensus and Species Use Value

The medicinal plants that are presumed to be effective in treating a certain disease have higher ICF values. Table 5 shows disease categories with relatively higher ICF values: 'mich' and febrile diseases (0.80), evil eye and satan beshita (devil sickness) (0.70), and respiratory and throat infections (0.64). This may indicate high incidence of these types of diseases in the region, possibly due to the poor socio-economic and sanitary conditions of the people. The categories of diseases that are only treated by the healers and those that are rare have lower ICF values. These include swelling and cancer (38), and sensorial disease (0.25). The medicinal plants that are widely used by the local people have higher FL values than those that are less popular. On the other hand, medicinal plants that are known as remedies of a single aliment have 100% fidelity level than those that are used as remedies for more than one type of aliment. For example, *Plumbago zeylanicum *is used to treat cancer, respiratory infection, swelling, and rheumatic pain and its FL value is 40% (Table 6).

**Table 5:** ICF values of category of aliments

Category	**Species**	**(%) All Species**	**Use citations**	**(%) All use citations**	**ICF value**
'Mich' and febrile diseases	6	9%	26	11%	0.80
Evil eye and satan beshita	13	20%	41	18%	0.70
Respiratory and throat infections	6	9%	15	7%	0.64
Rabies and internal disease	17	26%	45	20%	0.64
Gastrointestinal disorder and parasites infections	23	35%	60	26%	0.63
Venereal disease and impotence	7	11%	13	6%	0.50
External injuries and parasites infections	19	29%	33	14%	0.44
Snake bite	4	6%	6	3%	0.40
Swelling and cancer	9	14%	14	6%	0.38
Sensorial disease	4	6%	5	2%	0.25

**Table 6:** FL value of medicinal plants

**Species and Family**	Local name	Therapeutical uses	**Fidelity level (FL)**
*Carissa spinarum *L. Apocynaceae	Agam	evil eye	100%
*Clausena anisata *(Willd.) Benth Rutaceae	Limbche	evil eye	100%
*Acokanthera schimperi *(A. DC.) Schweinf. Apocynaceae	Yemerz Enchet	'kusil, yetat merz'	100%
*Calpurnia aurea *(Alt.) Benth. Fabaceae	Digita	diarrhea	100%
*Ficus thonningii *Blume. Moraceae	Chibha	'ayn bar teza'	100%
*Cyphostemma junceum *(Webb) Decoings ex Wild & Drummond Vitaceae	Etse Zewe	snake bite	100%
*Sansevieria erythraeae *Mattei Dracaenaceae	Algeti/chiret	'sinfete wesib'	100%
*Zehneria scabra *Asteraceae	Hareg Ressa (Este Sabek)	'mich', 'kintarot'	86%
*Stephania abyssinica *(Dillon. & A. Rich.) Walp. Menispermaceae	Kib Kitel/Etse Eyesus	stomachache/'kuruba', babies' sickness	80%
*Phytolacca dodecandra *L'Herit Phytolaceae	Endod	'wef beshita', 'kusil'	75%
*Verbena officinalis *L. Verbenaceae	Atuch	stomachache, evil eye, snake bite	73%
*Ocimum lamiifolium *Hochst. Lamiaceae	Dama Kesse	'mich', 'kusil'	67%
*Croton marcostachyus *Del. Euphorbiaceae	Bissana	gastrointestinal disorder, 'wef beshita'	63%
*Justicia schimperiana *(Hochst. ex A. Nees) T. Anders Acanthaceae	Sensel (Smiza)	evil eye, 'wef beshita', 'kuruba'	63%
*Capparis tomentosa *Lam. Capparidaceae	Gumero	evil eye, 'satan beshita, 'tesbo beshita'	57%
*Cucumis ficifolius *A. Rich. *Curcurbitaceae*	Yemidir Embuay	stomachache, 'kuruba', 'chiffea', 'majrat getr', 'nessr', rabies, 'wef beshita'	50%
*Plumbago zeylanicum *L. Plumbaginaceae	Amira	coughing, 'kurtimat', cancer, swelling	40%
*Dorstenia barnimiana *Schweinf. Moraceae	Work Bemeda	cancer, rabies, syphilis, 'wef beshita', 'yeahya kintarot', 'mushuro'	22%

## Declaration of competing interests

The author(s) declare that they have no competing interests.

## Authors' contributions

The authors have made substantive intellectual contributions to this study in data collection, identification of plants, preparation of the manuscript and proof reading.
